# High miR-451 expression in peripheral blood mononuclear cells from subjects at risk of developing rheumatoid arthritis

**DOI:** 10.1038/s41598-021-84004-3

**Published:** 2021-02-25

**Authors:** Klára Prajzlerová, Olga Kryštůfková, Petra Hánová, Veronika Horváthová, Monika Gregová, Karel Pavelka, Jiří Vencovský, Ladislav Šenolt, Mária Filková

**Affiliations:** 1 Institute of Rheumatology, Prague, 128 50 Czech Republic; 2 Department of Rheumatology, 1St Faculty of Medicine, Charles University, Prague, 128 00 Czech Republic; 3 Faculty of Science, Charles University, Prague, 128 00 Czech Republic

**Keywords:** Immunology, Rheumatology

## Abstract

Individuals carrying anti-citrullinated protein antibodies (ACPA) are considered at high risk of developing rheumatoid arthritis (RA). The altered expression of miRNAs contributes to the pathogenesis of RA. We aimed to identify differentially expressed miRNAs in the peripheral blood of ACPA-positive individuals with arthralgia at risk of RA compared to healthy controls (HC) and to determine their implications in the preclinical phase of RA. A comprehensive analysis of miRNAs revealed the dysregulation of miR-451 in peripheral blood mononuclear cells (PBMC) and plasma from RA-risk individuals. Higher miR-451 expression in PBMC from RA-risk individuals was further validated. Notably, miR-451 was previously shown to regulate CXCL16, a protein involved in RA pathogenesis. The expression of miR-451 in PBMC positively correlated with the CXCL16 mRNA, which could be secondary to the inflammation-induced expression of miR-451. Transfection of monocytes with pre-miR-451 in vitro resulted in the downregulation of CXCL16. Moreover, flow cytometry revealed a lower count of CXCL16-positive monocytes in RA-risk individuals. We propose that the constitutive or inflammation-induced upregulation of miR-451 in PBMC downregulates the expression of CXCL16, reduces the inflammatory milieu and thereby strives to delay the shift from the preclinical phase to the clinical manifestation of RA. This hypothesis warrants further investigation.

## Introduction

Rheumatoid arthritis (RA) is a chronic autoimmune inflammatory disease that is typically characterized by joint destruction and deformities. The presence of autoantibodies, such as anti-citrullinated protein antibodies (ACPA) or rheumatoid factor (RF), may precede the clinical manifestation of RA by many years^1^. The pathogenesis of RA begins in asymptomatic individuals with certain genetic or environmental risk factors for RA that cause the development of systemic autoimmunity. This asymptomatic phase is followed by arthralgia without clinical arthritis and finally by the manifestation of clinically detectable arthritis^2^. Classification criteria for RA published in 2010 enabled the early recognition of the disease^3^. Since individuals with both ACPA and RF have a 10.5 times higher risk of developing RA than seronegative individuals^4^, researchers are currently focused on targeting individuals at risk of developing RA (the preclinical phase of RA) to delay or even prevent the onset of RA^5^.

MicroRNAs (miRNAs) are small non-coding RNAs that, depending upon base pairing to messenger RNA (mRNA), mediate mRNA cleavage, translational repression or mRNA destabilization. The altered expression of miRNAs and subsequent dysregulation of their target genes have been shown to contribute to the pathophysiology of many autoimmune diseases, including RA^6^. In addition to their intracellular expression, miRNAs are present in body fluids in a stable form that is protected from endogenous RNase activity^7^. Extracellular miRNAs are actively secreted in microvesicles or incorporated into complexes with Argonaute 2 and high-density lipoproteins^8–10^ Therefore, extracellular miRNAs may represent a versatile communication tool, and their accessibility in body fluids or the expression signature in the tissue makes them potential clinical biomarkers^6^.

The aim of the present study was to identify a differentially expressed miRNA in the peripheral blood from individuals with arthralgia at risk of developing RA compared to healthy controls (HC) and to determine its potential implications in the preclinical phase of RA.

## Results

### Clinical characteristics

**Figure 1:**
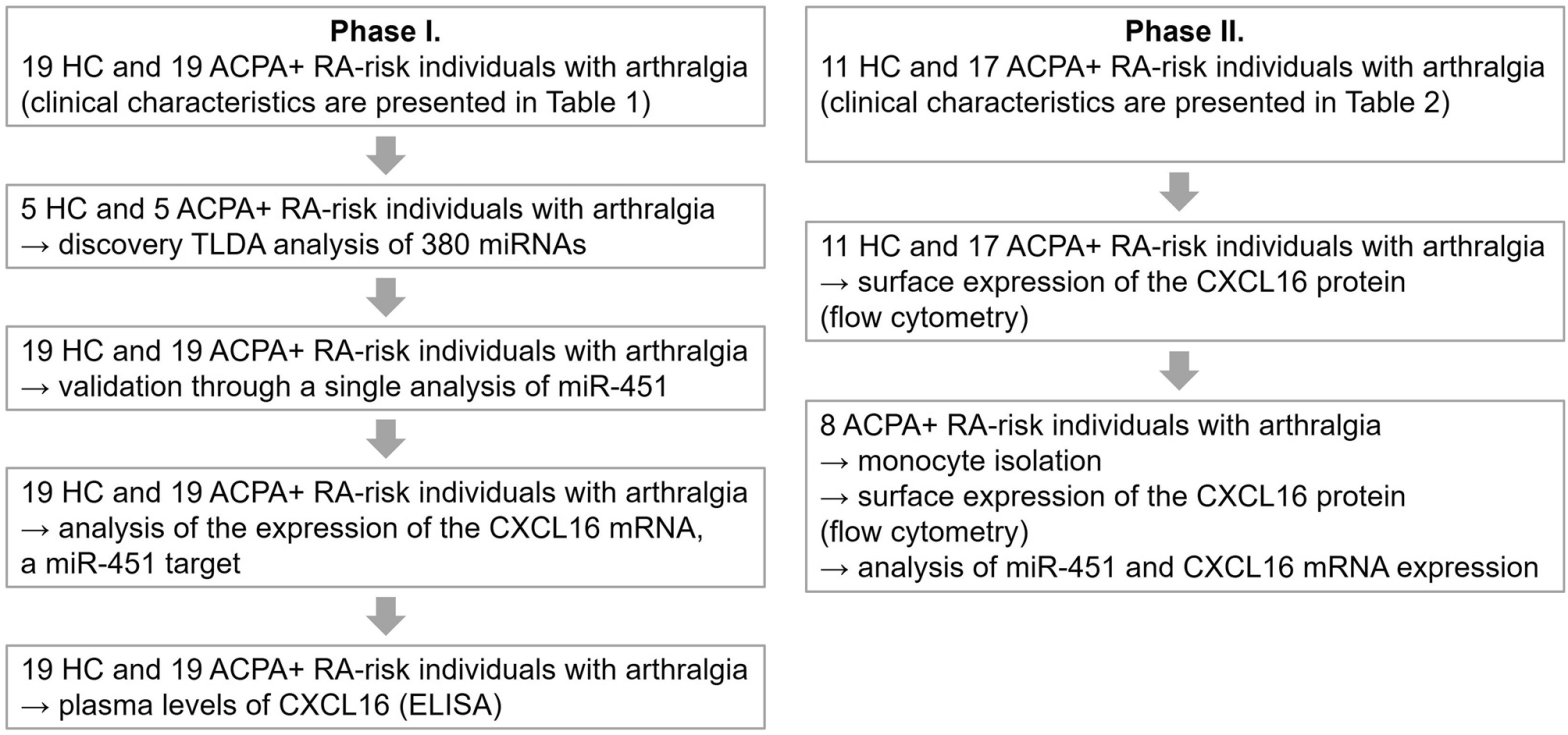
Workflow of our study. In Phase I, miRNAs in plasma and PBMC from ACPA-positive individuals with arthralgia at risk of developing RA and healthy controls (HC) were analysed. Phase II included a further analysis of target genes of selected miRNAs.

The study was divided into Phase I and Phase II, and the workflow is presented in Fig. 1.

Our Phase I study included 19 RA-risk individuals with arthralgia; all of these patients were ACPA-positive (Table 1). The median duration of arthralgia at inclusion was 18.38 [13.80–49.79] months. All RA-risk individuals were followed prospectively, with a median follow-up of 43.17 [28.58–96.54] months, and 6 individuals were lost to follow-up. Five RA-individuals developed arthritis within 39.67 [23.77–66.04] months of follow-up, with CRP levels of 3.15 [1.14–22.07] mg/l and a DAS28-CRP score of 3.41 [2.53–4.50] at the time of arthritis manifestation.

**Table 1:** Clinical characteristics of ACPA-positive individuals with arthralgia at risk of RA and healthy controls (Phase I).

Variable	RA-risk individuals	HC	*p* Value
n	19	19	–
Gender, female/male	13/6	12/7	NS
Age, years	39.72 [33.16–48.13]	43.00 [31.92–58.88]	NS
CRP, mg/l	2.70 [0.87–6.93]	1.42 [0.54–2.20]	0.05
ESR, mm/h	11.00 [6.00–19.00]	*	–
VAS, mm	10.00 [0.00–50.00]	NA	–
TJC, n	0.00 [0.00–1.00]	NA	–
RF positivity, n (%)	12 (63%)	0 (0%)	< 0.001
ACPA positivity, n (%)	19 (100%)	0 (0%)	< 0.001
Anti-CCP, U/ml	91.00 [34.70–800.00]	15.13 [13.42–17.30]	0.002
Anti-MCV, U/ml	41.40 [20.90–199.50]	8.20 [5.9–9.80]	< 0.001

Abbreviations: ACPA, anti-citrullinated protein antibodies (positive for at least one of the following antibodies: anti-CCP or anti-MCV); CRP, C-reactive protein; ESR, erythrocyte sedimentation rate; HC, healthy controls; NA, not applicable; NS, not significant; RF, rheumatoid factor (positivity of at least one of the following: IgG, IgA, or IgM RF assessed using an ELISA); TJC, 68 tender joint count; VAS, visual analogue scale (patient’s global health assessment). Data are presented as medians and interquartile ranges [IQRs]. *Missing data.

Our Phase II study involved 17 RA-risk individuals with arthralgia that were not included in Phase I (Table 2). The median duration of arthralgia in ACPA-positive RA-risk individuals at inclusion in Phase II was 48.43 [24.59–93.45] months. All RA-risk individuals were followed prospectively, with a median follow-up of 31.20 [22.54–34.29] months, and 2 individuals were lost to follow-up. Two individuals developed arthritis within 16.61 [8.91–24.30] months of follow-up, with CRP levels of 31.54 [14.50–48.57] mg/l and a DAS28-CRP score of 4.79 [4.71–4.87] at the time of arthritis manifestation.

**Table 2:** Clinical characteristics of ACPA-positive individuals with arthralgia at risk of RA and healthy controls (Phase II).

Variable	RA-risk individuals	HC	*p* Value
n	17	11	–
Gender, female/male	15/2	11/0	NS
Age, years	45.55 [39.54–53.15]	39.67 [31.26–47.82]	NS
CRP, mg/l	2.69 [1.25–3.46]	*	–
ESR, mm/h	9.00 [5.50–11.50]	*	–
VAS, mm	10.00 [0.00–25.00]	NA	–
TJC, n	0.00 [0.00–2.50]	NA	–
RF positivity, n (%)	6 (35%)	1 (9%)	NS
ACPA positivity, n (%)	17 (100%)	0 (0%)	< 0.001
Anti-CCP, U/ml	664.00 [37.40–744.30]	13.48 [12.38–15.81]	< 0.001
Anti-MCV, U/ml	31.10 [10.10–338.9]	7.70 [6.08–10.70]	0.006

### Identification of the differential expression of miR-451

First, we employed a comprehensive discovery analysis of miRNAs in plasma and peripheral blood mononuclear cells (PBMC) from 5 ACPA-negative healthy controls (HC) and 5 ACPA-positive RA-risk individuals with arthralgia (3 developed arthritis within 62.25 [15.29–69.83] months of follow-up). Subjects included in this discovery analysis were randomly selected from all subjects participating in the Phase I study. None of the patients had any clinical evidence of arthritis at the time of inclusion in the study or a medical history of musculoskeletal, inflammatory or autoimmune conditions. The clinical characteristics of these selected individuals were not significantly different from all Phase I subjects.

This comprehensive analysis of 380 miRNAs in plasma and PBMC was performed using Human Pool A TaqMan Low-Density Array (TLDA). Of the 380 analysed miRNAs, 231 miRNAs were detected in PBMC from at least 1 RA-risk individual with arthralgia, and 225 miRNAs were detected in at least 1 HC. For comparison, 125 extracellular circulating miRNAs were detected in the plasma of at least 1RA-risk individual with arthralgia, and 124 circulating miRNAs were detected in at least 1 HC sample. No miRNA was expressed in any of the five samples in the patient group and was completely absent in the comparator group. Only miRNAs expressed in all plasma or PBMC samples at a Ct cycle of less than 30 with more than a 1.5-fold difference between the groups were predefined for inclusion in the next validation step.

**Figure 2:**
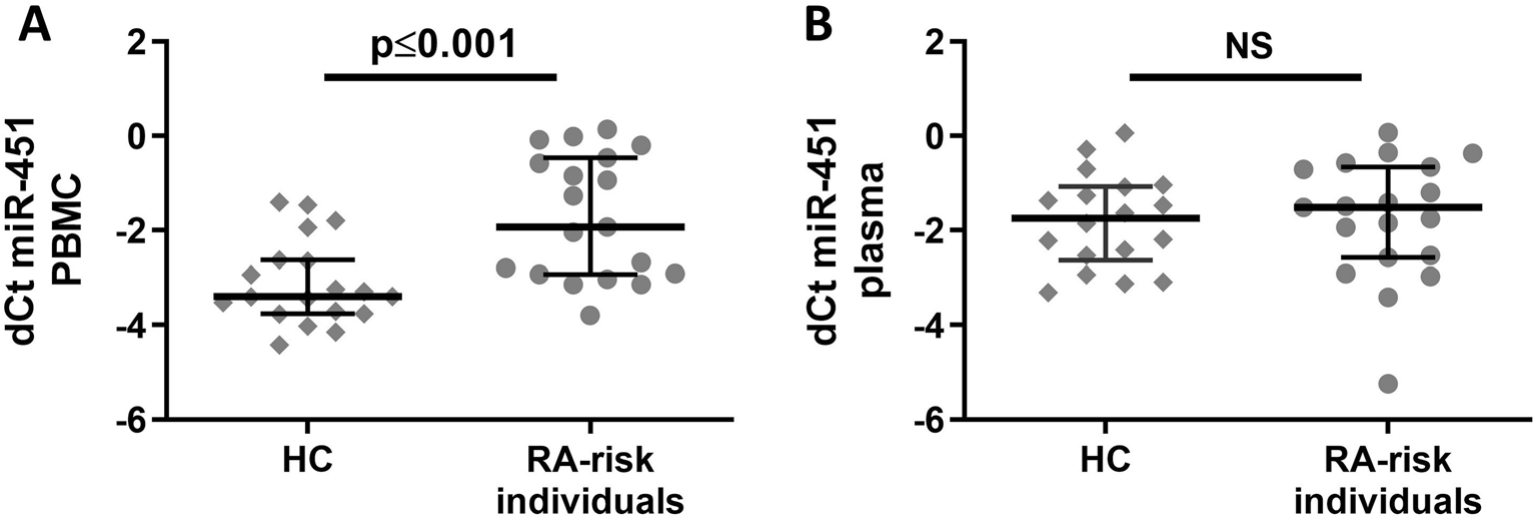
Expression of miR451 in (**A**) PBMC and (**B**) plasma from healthy controls (HC) and RA-risk individuals with arthralgia in the validation cohort using single assays. *NS, not significant*.

Of all tested miRNAs, only miR-451 fulfilled these predefined criteria. The TLDA analysis revealed 2.43-fold higher expression of miR-451 in PBMC (*p* = 0.199) and 1.55-fold lower levels of circulating miR-451 (*p* = 0.606) in RA-risk individuals with arthralgia compared to HC. Next, single assay validation in larger Phase I cohorts (n = 19 individuals in each group, clinical characteristics are provided in Table 1) confirmed the 3.19-fold higher expression of miR-451 in PBMC from RA-risk individuals with arthralgia compared to HC (*p* ≤ 0.001). However, the plasma levels of circulating miR-451 were comparable between the groups (*p* = 0.968) (Fig. 2).

The expression of miR-451 in PBMC from RA-risk individuals with arthralgia positively correlated with the visual analogue scale (VAS) score of the patient’s global health (r = 0.484; *p* = 0.036), and a trend towards an association with the tender joint count (TJC) (r = 0.401; *p* = 0.089) was observed. Nonetheless, no correlations with inflammatory markers, the erythrocyte sedimentation rate (ESR) (r = − 0.043; *p* = 0.869) or C-reactive protein (CRP) levels (r = 0.238; *p* = 0.328) were observed.

No associations were observed between the plasma levels of circulating miR-451 with the VAS score (r = − 0.071; *p* = 0.773), TJC (r = 0.010; *p* = 0.967), ESR (r = − 0.413; *p* = 0.099), CRP level (r = − 0.162; *p* = 0.507) or with the expression of miR-451 in corresponding PBMC samples.

### Analysis of CXCL16 as a potential miR-451 target gene

Given the dysregulation of miR-451 in PBMC from ACPA-positive RA-risk individuals, we searched for potential miR-451 target genes with a plausible role in the development of RA. Prediction algorithms based on potential complimentary binding sites and in vitro gene reporter assays confirmed that CXCL16 is a direct target of miR-451^11^. This molecule is involved in RA pathogenesis^12^ and was therefore taken subjected to further analysis.

**Figure 3:**
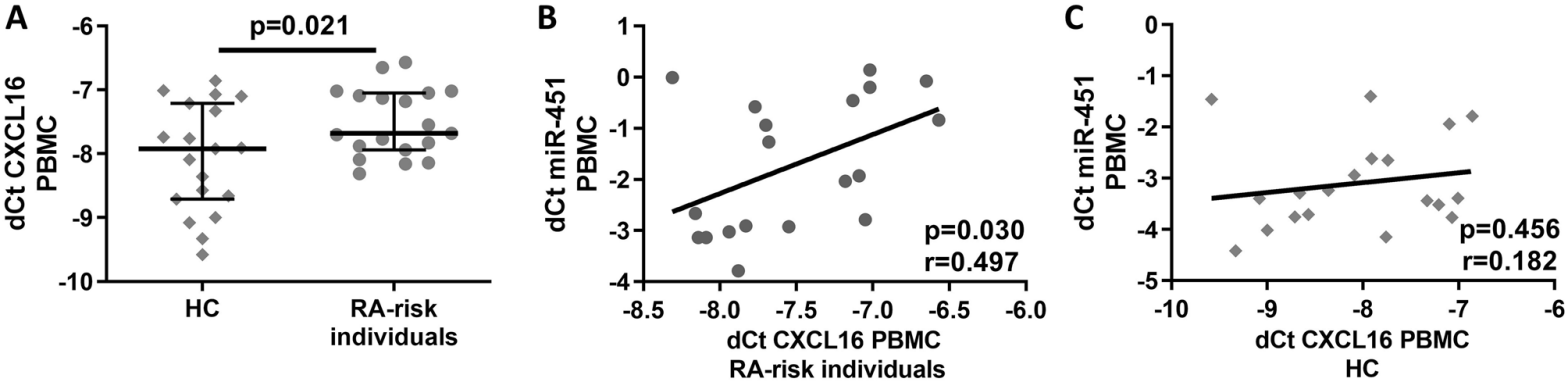
(**A**) Higher expression of the CXCL16 mRNA in PBMC from RA-risk individuals with arthralgia compared to HC. (**B**) A positive correlation between CXCL16 and miR-451 expression in PBMC from RA-risk individuals with arthralgia was observed. (**C**) CXCL16 expression was not correlated with miR-451 expression in PBMC from HC.

At the mRNA level, CXCL16 was expressed at 1.47-fold higher levels in PBMC from RA-risk individuals with arthralgia compared to HC (*p* = 0.021) (Fig. 3A) in Phase I cohort and positively correlated with the TJC (r = 0.576; *p* = 0.010), but not with the VAS score (r = 0.126; *p* = 0.606), ESR (r = − 0.084; *p* = 0.749) or CRP level (r = 0.018; *p* = 0.943). The expression of the CXCL16 mRNA positively correlated with miR-451 expression (r = 0.497; *p* = 0.030, Fig. 3B) in RA-risk individuals with arthralgia, but a correlation was not observed in HC (r = 0.182; *p* = 0.456) (Fig. 3C).

The CXCL16 protein exists in 2 forms: a secreted form present in plasma or serum and a cell membrane-bound protein^13^.

**Figure 4:**
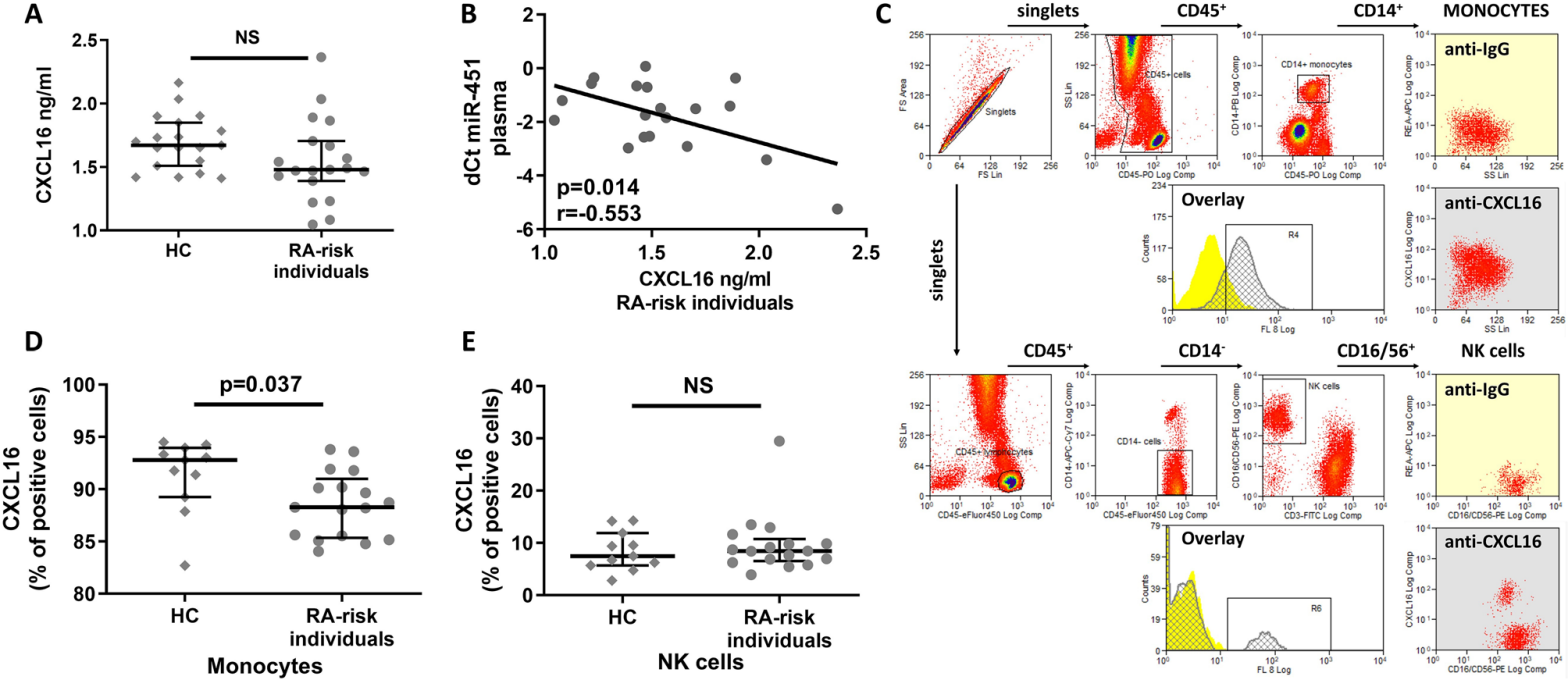
Analysis of CXCL16 levels in PBMC and plasma. (**A**) Plasma levels of CXCL16 were not different between HC and RA-risk individuals. (**B**) The negative correlation between plasma levels of CXCL16 and circulating miR-451 levels in RA-risk individuals with arthralgia. (**C**) Flow cytometry gating strategy and identification of the transmembrane form of CXCL16 in monocytes and NK cells. (**D**) Lower count of CXCL16-positive monocytes in RA-risk individuals with arthralgia compared to HC. (**E**) No differences in the numbers of CXCL16-positive NK cells were observed between RA-risk individuals with arthralgia and HC. *NS, not significant*. *The gating strategy was created in Summit V4.3.01 software (DAKO Cytomation, Fort Collins, CO, USA).*

In plasma, the levels of CXCL16 measured using an ELISA were not different between RA-risk individuals with arthralgia (n = 19) and HC (n = 19, *p* = 0.110) (Fig. 4A) in Phase I cohort, whose clinical characteristics are shown in Table 1. We identified a negative correlation between plasma CXCL16 levels and circulating miR-451 levels (r = − 0.553, *p* = 0.014) (Fig. 4B) and a positive correlation with the ESR (r = 0.570, *p* = 0.017) and CRP levels (r = 0.599, *p* = 0.007), but not with the VAS score (r = 0.078; *p* = 0.750) or TJC (r = − 0.288; *p* = 0.234) in RA-risk individuals with arthralgia. In contrast, we did not find a significant correlation between plasma levels of CXCL16 and circulating miR-451 (r = 0.257; *p* = 0.336) or CRP (r = 0.408, *p* = 0.083) in HC.

Next in Phase II, we further analysed the expression of the transmembrane protein and identified which cell subsets among PBMC expressed CXCL16. A flow cytometry analysis of different leucocyte subpopulations revealed that CXCL16 was expressed on the surface of monocytes and natural killer (NK) cells (Fig. 4C), but not on the surface of T and B lymphocytes. Moreover, a lower count of CXCL16-positive monocytes was observed in RA-risk individuals with arthralgia than in HC (*p* = 0.037, Fig. 4D), while no difference in the number of CXCL16-positive NK cells was observed (*p* = 0.696, Fig. 4E).

### Association between CXCL16 and miR-451 expression in monocytes

These differences in the levels of the CXCL16 protein in monocytes prompted us to further analyse the association between miR-451 and the expression of the CXCL16 mRNA and protein in isolated monocytes (n = 8). Unfortunately, a correlation was not observed between miR-451 and the CXCL16 mRNA (r = − 0.084, *p* = 0.825). Similar to the association between miR-451 and CXCL16 in PBMC mentioned above, the transcriptional data point towards either indirect regulation of CXCL16 by miR-451 or an additional regulatory mechanism in PBMC from RA-risk individuals with arthralgia. We explored these regulatory mechanisms by conducting proof-of-concept experiments in vitro.

**Figure 5:**
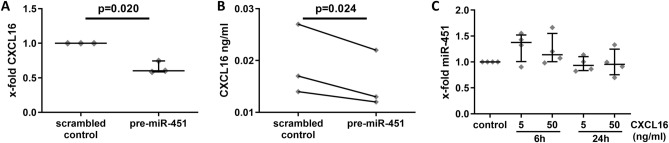
Regulation of miR-451 and CXCL16 in monocytes. Transfection of monocytes with pre-miR-451 downregulates the expression of CXCL16 at the (**A**) mRNA and (**B**) protein levels. (**C**) Stimulation of monocytes with CXCL16 tends to upregulate miR-451.

We first transfected monocytes (n = 3) with pre-miR-451 and observed the downregulation of CXCL16 at both the mRNA (*p* = 0.020) (Fig. 5A) and protein levels in cell culture supernatants (*p* = 0.024) (Fig. 5B). Next, we analysed whether CXCL16 regulates the expression of miR-451 in monocytes. Stimulation of monocytes (n = 4) with recombinant CXCL16 tended to upregulate miR-451 in monocytes in vitro after 6 h of stimulation (*p* = 0.250 for both concentrations) (Fig. 5C).

## Discussion

Roles for miRNAs in the development and maintenance of many autoimmune diseases, including RA, have been reported^6^. The identification of differentially expressed miRNAs in individuals with arthralgia at risk of progression to RA may provide a new perspective on the role of miRNAs in the development of RA. Recently, the first study on miRNAs in ACPA-positive individuals with arthralgia at risk of developing RA showed that serum levels of miR-22, miR486-3p, and miR-382 in these RA-risk individuals were associated with progression from systemic autoimmunity to clinical arthritis^14^. Here, we document the differential expression of miR-451 in PBMC from ACPA-positive RA-risk individuals with arthralgia compared to HC and its regulation of chemokine CXCL16.

Validation of our preliminary data from the comprehensive discovery miRNA analysis confirmed higher expression of miR-451 in PBMC from ACPA-positive RA-risk individuals with arthralgia compared to HC. The upregulation of miR-451 in PBMC might be either constitutive and represent a biomarker of the preclinical phase or might be increased in response to mild systemic inflammation, as evidenced here by higher CRP levels and supported by in vitro stimulation with CXCL16. However, we did not verify any difference in miR-451 expression in plasma samples from our validation cohort, although circulating miR-451 expression was previously shown to be increased in patients with established RA^15^. This discrepancy might be explained by the established clinical arthritis in patients with an advanced phase of RA. The lack of correlation between miR-451 expression in PBMC and plasma is not surprising, as extracellular circulating miRNAs may also originate from cells/organs other than PBMC, and cells may employ different priming or secretion mechanisms for various miRNAs^9^.

The overexpression of miR-451 in T cells and the correlation with the Disease Activity Score of 28 joints (DAS28), ESR, or IL-6 level was previously reported in treatment-naïve patients with early RA and patients with established RA compared to HC^16^. Since individuals with arthralgia at risk of developing RA do not have clinically defined arthritis, DAS28 is not applicable in our study population. A composite measure of “activity” in individuals at risk of RA is not available, and the global VAS score or even TJC might be considered a subjective assessment of discomfort in these individuals. ACPA antibodies induce joint pain in murine models^17^. Notably, miR-451 is also involved in the mechanism regulating pain^18^. We hypothesize that the correlation of miR-451 expression in PBMC with the VAS score may therefore reflect an unknown mechanism by which miR-451 regulates pain in ACPA-positive individuals with arthralgia.

The anti-inflammatory and anti-migratory effects of miR-451 mediated by the suppression of IL-6, TNF-α, or RANTES expression have been described in vitro in dendritic cells^19^ and synovial fibroblasts^20^ and in vivo in mice with collagen-induced arthritis^21^. Based on these findings, miR-451 potentially represents a biomarker with a functional role in inflammation or chemotaxis in individuals with RA, and thus we were interested in identifying the potential target genes of miR-451 with implications in the preclinical phase of RA. Based on the literature search, we selected CXC-motif ligand (CXCL) 16 as a gene of interest. Moreover, prediction algorithms and in vitro gene reporter assays confirmed that CXCL16 is a direct target of miR-451^11^.

Unexpectedly, we found a positive correlation between miR-451 and the CXCL16 mRNA in PBMC and a lack of correlation in monocytes from RA-risk individuals with arthralgia. Although our proof-of-concept in vitro experiments supported a direct regulatory mechanism, protein translation may also be reduced by partial binding of the miRNA to the mRNA without mRNA decay^22^, which might explain this positive correlation. In addition, these data suggest the presence of additional and more complex regulatory mechanisms in vivo*.* Different stimuli, such as increased systemic inflammation, upregulate miR-451 in the preclinical phase of RA, as shown here by the induction of miR-451 expression by CXCL16 in vitro. We admit that CXCL16 expression is also regulated by other miRNAs or triggers of different origins.

CXCL16 is a chemokine that is synthesized as a transmembrane protein by dendritic cells, macrophages, B and T lymphocytes, endothelial cells, and RA synovial fibroblasts or is cleaved by ADAM-10 and subsequently secreted in a soluble form^12,13,23–27^.

The data on serum CXCL16 levels in patients with RA and HC remain inconsistent^12,26,28^. Our data did not reveal a difference in plasma CXCL16 levels between individuals with arthralgia at risk of RA and HC; however, higher CRP levels and the positive correlation between CXCL16 with the CRP levels and ESR shown here in RA-risk individuals with arthralgia may reflect an inflammatory milieu in the preclinical phase. Similar to miR-451, CXCL16 may be secreted into the plasma from various cells, and the lack of a difference between our groups does not reflect the lack of regulation by miR-451 at the cellular level.

Consistent with the in vitro transfection experiments, the flow cytometry analysis of the CXCL16 protein in leukocyte subpopulations revealed lower transmembrane CXCL16-positive monocyte counts in RA-risk individuals with arthralgia compared to HC. The transmembrane CXCL16 protein was previously shown to stimulate inflammation by interacting with inflammatory cells, including macrophages^29^.

While monocytes from patients with active, established RA showed similar surface expression of CXCL16 as HC^23^, CXCL16 was expressed at high levels locally in the synovial tissue and synovial fluid of patients with RA^12,24–26^. During inflammation in the joint, locally activated cells express increased levels of adhesion molecules and chemokines that increase the influx of monocytes into the joint^26,30^. Monocytes differentiate into macrophages by entering the synovial tissue, which further triggers CXCL16 expression and contributes to the upregulation of CXCL16 and ADAM-10 in the RA synovium^26^. These changes result in increased concentrations of cleaved CXCL16 in the synovial fluid and the recruitment of CXCL16 receptor (CXCR6+)-expressing T cells to the synovial tissue, contributing to the inflammatory cascade associated with RA^24,26^. We hypothesize that the immune system of ACPA-positive individuals at risk of developing RA strives to reduce inflammation by upregulating miR-451, which subsequently downregulates CXCL16 expression in monocytes and thereby delays the shift from the preclinical phase to the clinical manifestation of arthritis.

Our study has several limitations. As mentioned above, the expression of CXCL16 in PBMC/monocytes is also regulated by miRNAs other than miR-451 or other stimuli. We are also aware that the local expression of CXCL16 in inflammatory cells present in the synovial tissue may not necessarily correspond with the expression of CXCL16 in peripheral blood leukocytes described here. Similar immunohistochemical features of synovial biopsy samples between ACPA-positive individuals without arthritis at risk of RA and controls (unlike patients with RA) have been reported^31^. However, the expression of CXCL16 in the synovial tissue of individuals in the preclinical phase of RA has not yet been shown. Since we are unable to obtain synovial fluid and synovial tissue from individuals at risk of RA who do not yet present with arthritis, we are unable to directly verify our hypothesis of the protective effect of miR-451 mediated by CXCL16 on the development of RA at the site of inflammation in the synovium. Additionally, we performed a cross-sectional analysis, and the comparison between the preclinical phase and the time of manifestation of clinical arthritis is missing. Only long-term follow-up and the collection of longitudinal data, including the collection of fresh samples from large cohorts of subjects who develop clinical arthritis in the future would truly verify our hypothesis.

In conclusion, we suggest that the upregulation of miR-451 in PBMC is either constitutive or increased in response to mild systemic inflammation in the preclinical phase, as evidenced here by higher CRP levels and a positive correlation between CXCL16 and CRP levels. Subsequently, miR-451 downregulates the expression of CXCL16 in monocytes and reduces the inflammatory milieu in the preclinical phase of RA, thereby attempting to delay the shift from the preclinical phase to the clinical manifestation of arthritis. With the increasing cascade of inflammatory stimuli, this protective mechanism may become insufficient to prevent the manifestation of arthritis. This hypothesis, however, warrants further investigation.

## Methods

### Study population

Our study included ACPA-positive RA-risk individuals with arthralgia and HC. By definition, all RA-risk individuals with arthralgia had no clinical evidence of arthritis at the time of inclusion in the study or any defined systemic inflammatory rheumatic disease, and all individuals were ACPA positive. Individuals at risk of RA were defined as ACPA-positive if they were positive for either an antibody against the cyclic citrullinated protein (anti-CCP) or modified citrullinated vimentin (anti-MCV) (further details are provided in the Methods—Protein analysis). All HC were healthy employees and their family members with no history of musculoskeletal, inflammatory, or autoimmune conditions or cancer, and were all ACPA-negative.

Our initial analysis (Phase I, Fig. 1) included 19 ACPA-positive RA-risk individuals with arthralgia and 19 HC. The clinical characteristics are shown in Table 1.

The flow cytometry analysis (Phase II, Fig. 1) involved samples from 17 ACPA-positive RA-risk individuals with arthralgia and 11 HC included during further recruitment. No subject included in Phase II overlapped with the subjects involved in Phase I. The clinical characteristics are shown in Table 2. Significant differences were not observed in any clinical characteristics among individuals with arthralgia at risk of RA who were included in Phase I and Phase II.

All RA-risk individuals with arthralgia and HC were recruited from the outpatient clinic of the Institute of Rheumatology, Prague, Czech Republic. Written informed consent was obtained from all participants prior to enrolment. The study was approved by the local ethics committee at the Institute of Rheumatology in Prague. All experiments were performed in accordance with relevant guidelines and regulations.

### Analysis of miRNA and gene expression

Whole blood samples from all participants were collected in EDTA-containing tubes. All plasma samples were separated by centrifugation within 4 h of collection to ensure constant preanalytical conditions. PBMC were isolated by Ficoll–Paque density gradient centrifugation. CD14+ cells were labelled with magnetic StraightFrom Whole Blood CD14 MicroBeads and then separated using a Whole Blood Column Kit (both Miltenyi Biotec Inc., Gladbach, Germany). Plasma samples and cell culture samples were stored at − 80 °C. PBMC and freshly isolated monocytes were snap-frozen and stored at − 80 °C. No freeze–thaw cycles occurred before use.

### Monocyte culture and in vitro stimulation and transfection

For proof-of-concept experiments, monocytes were isolated from the blood of healthy donors using StraightFrom Whole Blood CD14 MicroBeads and Whole Blood Column Kit as described above. Cells were seeded in 24-mm diameter culture plates at a density of 7 × 10^5^ cells/well in RPMI 1640 medium (GIBCO, Grand Island, NY, USA) supplemented with 10% FBS (Lonza, Verviers, Belgium) and 1% Pen-Strep (Lonza).

For transfection experiments, cells seeded in 750 μl of RPMI 1640 (GIBCO) were transfected with Lipofectamine RNAiMAX Reagent using either Pre-miR miRNA Precursor Negative Control as a scrambled control or Pre-miR-hsa-miR-451a Precursor (30 pmol) (all Thermo Fisher Scientific, Waltham, MA, USA) according to the manufacturer’s instructions and incubated at 37 °C with an atmosphere containing 5% CO_2_ for 24 h. For stimulation experiments, the cells were stimulated with 5 or 50 ng of CXCL16 (R&D Systems, Inc., Minneapolis, MN, USA) or the corresponding control and incubated at 37 °C with an atmosphere containing 5% CO_2_ for 6 and 24 h. Cell culture supernatants were separated after centrifugation for 10 min and stored at − 80 °C until analysis.

### RNA isolation

Total RNA was extracted from plasma samples using phenol–chloroform, as previously described^32^. Plasma was homogenized with TRIzol LS reagent (Thermo Fisher Scientific), centrifuged at 12,000×*g* for 10 min at 4 °C and subjected to three cycles of acid phenol–chloroform (Thermo Fisher Scientific) extraction. RNA was precipitated by adding RNase-free glycogen (Roche Diagnostics, Mannheim, Germany) and 100% isopropanol, incubated at room temperature and centrifuged at 12,000×*g* for 10 min at 4 °C. The pellet was washed with 75% ethanol, and RNA was dissolved in RNase-free water. Three synthetic *C. elegans* miRNAs, cel-miR-39, cel-miR-54 and cel-miR-238 (Integrated DNA Technologies, Coralville, IA, USA), 25 fmol each, were spiked into plasma samples after denaturation and served as internal calibrators, as previously described^7^.

PBMC and freshly isolated and cell culture harvested monocytes were lysed with 700 µl of QIAzol Lysis Reagent (Qiagen, Düsseldorf, Germany). Total RNA was then isolated using the miRNeasy Mini Kit (Qiagen) according to the manufacturer’s instructions. RNA concentrations in all samples were measured using a NanoDrop 2000c spectrophotometer (Thermo Fisher Scientific).

### Analysis of miRNA expression

The expression of miRNAs was analysed as previously described^33^. First, total RNA was isolated from individual plasma and PBMC samples (5 samples from each group), and complementary DNA templates were obtained by reverse transcription using a TaqMan MicroRNA Reverse Transcription Kit and Megaplex RT Primers with equal amounts of RNA input. The cDNA templates were preamplified using 2× TaqMan PreAmp Master Mix and Megaplex PreAmp Primers (all Thermo Fisher Scientific) with a PCR thermocycler (Bio-Rad Laboratories, Hercules, CA, USA). A Human Pool A TaqMan Low-Density Array (TLDA) platform for microRNAs was selected for the discovery analysis of 380 miRNAs using the QuantStudio 7 Flex Real-Time PCR System (Thermo Fisher Scientific). All steps were performed according to the manufacturer’s instructions. The dCt method was used for relative quantification: dCt = Ct(array average) − Ct(miRNA of interest), followed by the x-fold change calculation.

Total RNA was reverse transcribed from the remaining non-pooled plasma and PBMC samples for subsequent single assay validation or from cultured monocytes using TaqMan Real-Time miRNA specific primers (including primers for cel-miR-39, cel-miR-54 and cel-miR-238 in plasma or RNU44 in PBMC and monocytes that were used for normalization) and then amplified by Real-Time PCR with TaqMan probes and TaqMan Universal PCR Master Mix using the QuantStudio 7 Flex Real-Time PCR System (all Thermo Fisher Scientific). The dCt method was used for relative quantification: dCt = Ct(spike-in average or RNU44) − Ct(miRNA of interest). Lower dCt values represent lower expression levels of particular miRNAs. All data were analysed with QuantStudio 7 Flex Real-Time PCR System Software (Thermo Fisher Scientific).

### Analysis of protein-coding gene expression

Total RNA was reverse transcribed from PBMC or monocytes using a High-capacity cDNA Reverse Transcription kit, and PCR was performed using a standard protocol with a QuantStudio 7 Flex Real-Time PCR System. Predeveloped TaqMan probes were used to detect CXCL16 and beta-actin (ACTB), which was used for normalization (all Thermo Fisher Scientific). The dCt method was used for relative quantification as follows: dCt = Ct(ACTB) − Ct(studied gene). Higher dCt values represent higher expression levels.

### Protein analysis by ELISA

The levels of CXCL16 in plasma and supernatant samples were measured using a commercially available ELISA kit (Human CXCL16 Quantikine ELISA, R&D Systems, Inc.) according to the manufacturer’s protocol. Rheumatoid factor IgA, IgG, IgM and anti-CCP titres were measured using commercial ELISAs (TestLine Clinical Diagnostics s.r.o.) with a cut-off of 22.0 U/ml and anti-MCV titres were measured using an ELISA (Orgentec Diagnostika GmbH, Mainz, Germany) with a cut-off of 19.9 U/ml. Absorbance at a primary wavelength of 450 nm was detected using a Sunrise ELISA reader (Tecan Group Ltd., Zürich, Switzerland), and the data were quantified using Kim version 5.43.01 software (Daniel Kittrich—Software Production, Prague, Czech Republic).

### Flow cytometry analysis

Whole blood samples from both groups were collected in EDTA-containing tubes. The presence of CXCL16 on the surface of cells was analysed by performing direct staining of 100 µl of whole blood or 50 µl of isolated monocytes resuspended in Whole Blood Column Elution Buffer (Miltenyi Biotec Inc.). After 20 min of incubation with conjugated antibodies for membrane staining (Supplementary Table 1), whole blood samples were lysed with a 1 × BD Lysing solution (Becton Dickinson), and all samples were washed twice with PBS (Thermo Fisher Scientific).

Monocytes were defined as CD45^+^CD14^+^ cells. Lymphocyte subpopulations (CD45^+^CD14^−^) were defined as CD19^+^CD3^−^ B lymphocytes, CD3^+^ T lymphocytes and CD16/56^+^CD3^−^ NK cells. Surface expression of CXCL16 in leukocyte populations was compared to the universal isotype control (REA Control (S)-APC) staining (Miltenyi Biotec Inc.). The gating strategy is provided in Fig. 4A. A list of antibodies used for the flow cytometry analysis is provided in Supplementary Table 1.

Stained samples were quantified using a CyAnADP flow cytometer (DAKO Cytomation, Fort Collins, CO, USA). The stable setting of photomultipliers was preserved with the daily setting calibrated on Rainbow Calibration Particles (SPHERO Rainbow Calibration Particles, Spherotech, Lake Forest, IL, USA).

### Statistical analysis

Data are presented as medians and interquartile ranges [IQRs]. Two-sample T tests or Mann–Whitney tests were used for comparisons between 2 groups, and Fischer’s exact test was used where appropriate. Pearson’s or Spearman’s correlation coefficients were calculated to determine the correlation between any two variables. P values less than 0.05 were considered statistically significant. All analyses were performed and graphs were generated using GraphPad Prism 6 software (GraphPad Software, La Jolla, CA, USA).

## Supplementary Information

Supplementary Table
