# Supplementary material for: High miR-451 expression in peripheral blood mononuclear cells from subjects at risk of developing rheumatoid arthritis
Source: Sci Rep. 2021 Feb 25;11:4719. doi: 10.1038/s41598-021-84004-3 (PMC7907058; doi:10.1038/s41598-021-84004-3)
Supplement: Supplementary file 1 — Supplementary Table [file 41598_2021_84004_MOESM1_ESM.docx]

**Supplementary Table 1** List of antibodies used for flow cytometry analysis

| Antibody | Fluorochrome | Clone | Company (catalogue number) |
| --- | --- | --- | --- |
| CD3 | FITC | UCHT1 | DAKO, Agilent, Santa Clara, CA, USA (F081801) |
| CD16 | PE | 3G8 | Beckman Coulter, Brea, CA, USA (A07766) |
| CD56 | PE | N901 | Beckman Coulter, Brea, CA, USA (A07788) |
| CD19 | PE-Cy7 | J3-119 | Beckman Coulter, Brea, CA, USA (IM3628) |
| CD45 | eFluor450 | 2D1 | Thermo Fisher Scientific, Waltham, MA, USA (9048-9459-120) |
|  | PO | HI30 | Exbio, Vestec, Czech Republic (PO-684-T100) |
| CD14 | APC-Cy7 | MφP9 | Beckton Dickinson, Franklin Lakes, NJ, USA (333951) |
|  | PB | M5E2 | Beckton Dickinson, Franklin Lakes, NJ, USA (558121) |
| CXCL16 | APC | REA873 | Miltenyi Biotec Inc.,Gladbach, Germany (130-114-237) |
| REA Control (S) | APC | REA293 | Miltenyi Biotec Inc.,Gladbach, Germany (130-104-614) |
